# Cross-cultural adaptation and validation of the French version of the Expanded Prostate cancer Index Composite questionnaire for health-related quality of life in prostate cancer patients

**DOI:** 10.1186/s12955-016-0571-y

**Published:** 2016-12-06

**Authors:** Amélie Anota, Anne-Sophie Mariet, Philippe Maingon, Florence Joly, Jean-François Bosset, Anne-Valérie Guizard, Hugues Bittard, Michel Velten, Mariette Mercier

**Affiliations:** 1 French National Quality of Life in Oncology Platform, Besançon, France; 2 Methodology and Quality of Life in Oncology Unit, University Hospital of Besançon, Besançon, France; 3 EA3181, University of Franche-Comté, Besançon, France; 4 Service biostatistiques et information médicale (DIM), University Hospital of Dijon, Dijon, France; 5 INSERM, CIC 1432, Centre d’investigation clinique, University Hospital of Dijon, Dijon, France; 6 INSERM UMR 1181 Biostatistiques, Biomathématiques, Pharmacoépidemiologie et Maladies infectieuses (B2PHI), University of Burgundy Franche-Comté, Dijon, France; 7 Department of Radiation Oncology, Georges François Leclerc Cancer Center, Dijon, France; 8 Department of Medical Oncology, Centre François Baclesse, Inserm U1086, Caen, France; 9 Registre général des tumeurs du Calvados, Cancers & Préventions - U 1086 Inserm - UCBN, Centre François Baclesse, Caen, France; 10 Department of Urology, University Hospital of Besançon, Besançon, France; 11 Registre des Cancers du Bas-Rhin, Strasbourg, France; 12 Université Pierre et Marie Curie, Paris, France

**Keywords:** Health-related quality of life, Cross-cultural adaptation, Validation, Psychometric properties, EPIC questionnaire, Prostate cancer, Classical test theory

## Abstract

**Background:**

Health-related quality of life (HRQoL) has been positioned as one of the major endpoints in oncology. Thus, there is a need to validate cancer-site specific survey instruments. This study aimed to perform a transcultural adaptation of the 50-item Expanded Prostate cancer Index Composite (EPIC) questionnaire for HRQoL in prostate cancer patients and to validate the psychometric properties of the French-language version.

**Methods:**

The EPIC questionnaire measures urinary, bowel, sexual and hormonal domains. The first step, corresponding to transcultural adaptation of the original English version of the EPIC was performed according to the back translation technique. The second step, comprising the validation of the psychometric properties of the EPIC questionnaire, was performed in patients under treatment for localized prostate cancer (treatment group) and in patients cured of prostate cancer (cured group). The EORTC QLQ-C30 and QLQ-PR25 prostate cancer module were also completed by patients to assess criterion validity. Two assessments were performed, i.e., before and at the end of treatment for the Treatment group, to assess sensitivity to change; and at 2 weeks’ interval in the Cured group to assess test-retest reliability. Psychometric properties were explored according to classical test theory.

**Results:**

The first step showed overall good acceptability and understanding of the questionnaire. In the second step, 215 patients were included from January 2012 to June 2014: 125 in the Treatment group, and 90 in the Cured group. All domains exhibited good internal consistency, except the bowel domain (Cronbach’s α = 0.61). No floor effect was observed. Test-retest reliability assessed in the cured group was acceptable, expect for bowel function (intraclass coefficient = 0.68). Criterion validity was good for each domain and subscale. Construct validity was not demonstrated for the hormonal and bowel domains. Sensitivity to change was exhibited for 5/8 subscales and 2/4 summary scores for patients who experienced toxicities during treatment.

**Conclusions:**

The French EPIC questionnaire seems to have adequate psychometric properties, comparable to those exhibited by the original English-language version, except for the construct validity, which was not available in original version.

## Background

Prostate cancer is the second most common cause of cancer and the sixth leading cause of cancer death among men worldwide, with an estimated 1.1 million cases in 2012 and 307,000 new deaths [1]. In France, prostate cancer has been the most frequent cancer in men for the last two decades, with around 54,000 new prostate cancers diagnosed in 2011 [2]. However, prostate cancer is the fifth overall cause of cancer-related death, with less than 10,000 deaths per year as of 2011 [2].

For prostate cancer that is diagnosed when still at a local stage, several curative treatment strategies exist that can achieve long-term remission. However, these treatments can induce significant functional impairment at the urinary, sexual, digestive and hormonal level [3, 4]. In this context, health-related quality of life (HRQoL) is an important endpoint for such patients. Moreover, HRQoL is now recognized as a second primary endpoint by the American Society of Clinical Oncology and the Food and Drug Administration if no effect of treatment on overall survival is observed [5–7].

In order to capture all symptoms specific to prostate cancer and the side effects of prostate cancer treatment, it is recommended to use disease-specific HRQoL questionnaires [8]. These questionnaires must also be adapted to the culture and the language of the study [9], especially in non-English-speaking countries, and validation of the psychometric properties of adapted questionnaires remains mandatory.

Few HRQoL questionnaires specific to prostate cancer have been validated in the French language. The QLQ-PR25 prostate-cancer-specific module of the European Organization of Research and Treatment of Cancer (EORTC) is available and validated in French [10], but only explores symptoms from a factual point of view. The University of California-Los Angeles Prostate Cancer Index (UCLA-PCI) is widely used in English-speaking countries [11]. The Expanded Prostate Cancer Index Composite (EPIC) was developed from the UCLA-PCI by supplementing it with items focusing on urinary irritative and obstructive voiding symptoms and with items addressing hormonal symptoms [12]. The availability of a validated French-language version of the EPIC, which would be better adapted to epidemiological studies [13], is thus essential to allow comparison of French data with existing reports in the international literature.

In this context, the objective of this study was to perform transcultural adaptation and validation of the French version of the EPIC questionnaire according to classical test theory. This study is part of the French National QALIPRO project that aims to investigate the long-term side effects of prostate cancer, in which the EPIC questionnaire is used.

## Methods

### Study design

The validation of the French version of the EPIC questionnaire was performed in two steps. Nine French cancer care centers and university hospitals participated in patient recruitment.

#### Step 1: Transcultural adaptation and qualitative validation (face validity)

Transcultural adaptation of the EPIC questionnaire in French was done using back translation technique and was pretested on a planned sample of 50 patients [14]. These patients were recruited during urology or radiotherapy consultations. Patients had to fill out a debriefing questionnaire to assess their completion of the EPIC: i.e., they could indicate if the questionnaire was too long, or too complicated, or if some items were found to be disturbing, irrelevant, redundant or missing, among other questions. The time required to complete the questionnaire was also recorded. This information gave an indication of both the quality of the translation and the possible acceptability of the questionnaire.

#### Step2: Quantitative validation of the psychometric properties of the questionnaire

##### Participants

For the second step, both patients with ongoing treatment and cured patients were recruited and categorized into two groups as follows:

- In the Cured group, patients had to be considered cured of prostate cancer (≥3 years after diagnosis), regardless of initial treatment, and attending a follow-up consultation. Patients with recurrence were excluded.
- In the Treatment group, patients were prospectively included before curative treatment for localized prostate cancer. Patients were eligible to participate if they had a histologically confirmed diagnosis of localized prostate cancer, and if they had no previous treatment for their prostate cancer. Patients were excluded if the curative treatment had already been performed, or in case of metastasis.

All patients had to have social security coverage, and had to be able to complete HRQoL questionnaires. All patients were fully informed of the study and provided signed written informed consent. The protocol was approved by the local ethics committee (Comité de Protection des Personnes Est II).

##### Questionnaires

Patients were required to complete the EPIC questionnaire, as well as the EORTC QLQ-C30 cancer specific questionnaire, and the QLQ-PR25 prostate cancer module, to assess criterion validity.

The EPIC questionnaire is a 50-item instrument specific to prostate cancer developed and validated in the English language [12]. Items are separated in domains and each item contains four or five response categories. The questionnaire assesses four domains, namely: urinary, bowel, sexual and hormonal; and each domain comprises two subscales, namely symptom severity (function subscale) and symptom-related impairment (bother subscale). The urinary domain can also be separated into two other subscales, combining both function and bother items, namely a urinary incontinence subscale and a urinary irritation/obstruction subscale. The last item evaluates overall satisfaction. Scale scores (one score per subscale) are transformed linearly to a 0-to-100 scale, with higher scores representing higher HRQOL, i.e., high function and low bother. A summary score is also generated for each domain, corresponding to the mean of the function and bother subscales. Scores were generated according to the recommendations of the questionnaire developers [15].

The QLQ-C30 includes 30 items and measures five functional scales (physical, role, emotional, cognitive and social functioning), global health status (GHS), financial difficulties and eight symptom scales (fatigue, nausea and vomiting, pain, dyspnea, insomnia, appetite loss, constipation, diarrhea) [16]. One score is generated for each dimension. Scores vary from 0 (worst) to 100 (best) for the functional dimensions and GHS, and from 0 (best) to 100 (worst) for the symptom dimensions, and were generated according to the EORTC Scoring Manual [17].

The QLQ-PR25 module contains 25 items assessing two functional scales (sexual activity and sexual functioning) and four symptomatic scales (urinary symptoms, bowel symptoms, hormone treatment-related symptoms and incontinence aid) specific to prostate cancer. This module must be completed in conjunction with the QLQ-C30 questionnaire. As with the QLQ-C30, one score is generated for each scale and standardized on a 0–100 scale such that a high score represents a high functional or symptomatic level [10].

##### Measurement times

Patients were required to complete the questionnaires twice, as follows:

- In the Cured group, patients completed the questionnaires at baseline (T_1_) and again 2 weeks later (T_2_) to assess test-retest reliability;
- In the Treatment group, patients completed the questionnaires immediately before the initiation of treatment (T_1_) and then again at the end of treatment (6 to 8 weeks after the first assessment, T_2_) to assess sensitivity to change.

### Sample size calculation

For the qualitative validation, we planned to include 50 patients.

For the quantitative validation of the psychometric properties of the EPIC questionnaire, it was planned to include 300 patients in order to ensure the statistical robustness of the analyses. In particular, exploratory factor analysis (EFA) to investigate the dimensionality of the questionnaire requires a minimum of 150 patients [18], with a minimum of 30 patients per response category with 5 response categories per item. We planned to include 100 patients in the Cured group and 200 patients in the Treatment group.

In the Treatment group, to highlight a minimal clinically important difference (MCID) of 10 points in one dimension between both questionnaire measurements, with a standard deviation (SD) of 20 points, a type I error of 5% and statistical power of 80%, a minimum of 150 patients was required (75 patients in two groups defined by an external criteria).

### Statistical analysis

Baseline socio-demographic and clinical characteristics of the patients are described using mean ± SD or median (range) for continuous variables, and number (percentage) for qualitative variables.

Face validity was assessed using the debriefing questionnaire from step 1.

All other analyses were performed on the validation population from step 2 including all patients (both Cured and Treatment groups) at the first measurement point (except if a specific population is concerned).

Acceptability of the questionnaire was assessed by the percentage of missing data (missing items and missing forms). A high proportion of missing forms may indicate poor acceptability of the instrument [8]. Information provided in the debriefing questionnaire in step 1 was also used to gain additional insights into the acceptability of the questionnaire. For example, if the patient found some questions to be disturbing, difficult to understand, or found the questionnaire too long, this could explain some missing data and could similarly lead to missing data in future studies. The debriefing questionnaire was summarized as number and percentage for each question for all patients of step 1. The mean ± SD time required to complete the questionnaire was also reported.

Floor and ceiling effects were estimated for each subscale and for the overall score. The number and percentage of patients who obtained the lowest or highest possible score for each subscale and overall domain were recorded. Floor or ceiling effects were considered to be present if more than 15% of the responders achieved the lowest or highest possible score respectively [19].

Reliability of the questionnaire was assessed using Cronbach’s α coefficient for internal consistency and the test-retest method for repeatability. Cronbach’s α was estimated for all subscales and overall domains. It was expected to be higher than 0.70 [20]. Test-retest reliability was assessed from the data of the Cured group with estimation of the interclass correlation coefficient (ICC) between assessments T_1_ and T_2_ [21].

Convergent and discriminant validities were evaluated using multitrait scaling analysis [18] conducted separately for the eight subscales and for the four domains. The convergent validity of each item was assessed using Spearman’s correlation coefficient between each item and its own subscale score, computed without including the corresponding item. The convergent validity was considered satisfactory if the correlation coefficient was higher than 0.40, in absolute value. For the discriminant validity, the correlation between each item and its own scale score was expected to be greater than the correlation between that item and the other scale scores. Similar analyses were conducted for each domain.

EFA was performed to assess the dimensionality of the questionnaire with orthogonal rotation of the factors and imposing four factors. All items were integrated into the EFA except the last item assessing overall satisfaction. EFA was performed on patients who had completed all items at the first measurement timepoint. The variance explained by the four factors was reported and the resulting factors were interpreted [22].

Criterion validity was assessed using a correlation matrix between the EPIC domain summary scores and the QLQ-PR25 scores. The correlation between the EPIC and QLQ-PR25 scores assessing the same HRQoL domain was expected to be higher than 0.4, in absolute value. Conversely, the correlation between each EPIC domain summary score and other scales of the QLQ-PR25 assessing other HRQoL domains was expected to be lower.

Sensitivity to change was assessed among patients from the Treatment group by comparing, using a paired *T*-test, the change in scores between T2 and T1 according to the presence or absence of at least one toxicity rated grade 2 or higher during treatment. We assumed that patients experiencing at least one toxicity would experience a greater deterioration in their HRQoL level than patients without toxicity. Mean change between T2 and T1, with the SD of the change between the two measures was reported. The Standardized Response Mean (SRM) was also estimated, corresponding to the mean change divided by the SD of change. An absolute SRM value less than 0.2 was considered as a “small” change, between 0.2 and 0.5 as “moderate”, and greater than 0.5 as “large” [23]. Each domain and subscale was analysed without any specific hypothesis as to whether the effect of toxicity on each scale was similar. The median (range) time between the two assessments was also reported. We expected a clinically significant change of at least 10 points in all the subscales and summary scores for patients who experienced toxicities during treatment.

All analyses were performed using SAS version 9.4 (SAS Institute Inc., Cary, NC, USA). All tests were two-sided and the type I error was set at 0.05. No adjustment for multiple testing was performed.

## Results

### Step1: Face validity

Forty-six patients were included in step 1. None of them considered the questionnaire to be too long. Five patients (11.6%) reported that they found at least one question disturbing. These patients mainly reported that the disturbing questions referred to symptoms that they did not experience, so they had some difficulty judging the impact of the given symptom on their HRQoL level, for example. Two patients mentioned that they found some questions to be disturbing, explaining that it was too private (questions 17 and 21 about sexuality). Four patients (8.7%) needed help completing the questionnaire. Twenty-eight patients (62.2%) considered that the questionnaire addressed relevant issues. Forty-one patients (91.1%) considered that the questionnaire might concern other men. Finally, 38 patients (84.4%) declared that the questionnaire enabled them to deal more easily with problems or difficulties related to their disease. The mean time required to complete the questionnaire was less than 20 min (mean 19.8, SD = 9.3). In light of these results, the French version of the EPIC questionnaire tested in step 1 was maintained as is for step 2.

### Step2: Quantitative validation of psychometric properties

#### Study population

In step 2, 215 patients were included between January 2012 and June 2014: 125 patients in the Treatment group and 90 patients in the Cured group. The mean age was 68 years (SD = 6.6). The baseline socio-demographic and clinical characteristics of the study population are summarized in Table 1.

**Table 1:** Baseline characteristics of the patients

	All patients		Cured group		Treatment group	
	*N* = 215		*N* = 90		*N* = 125	
	*N*	%	*N*	%	*N*	%
Age. mean (SD)	187	68.1 (6.6)	76	70.0 (6.5)	111	66.9 (6.3)
Marital status						
Living maritally	162	75.3	70	77.8	92	73.6
Divorced	17	7.9	6	6.7	11	8.8
Widower	6	2.8	3	3.3	3	2.4
Single	6	2.8	1	1.1	5	4.0
Missing data	24	11.1	10	11.1	14	11.2
Profession						
Retired	158	73.5	75	83.3	83	66.4
Professionally active	29	13.5	5	5.6	24	19.2
Unemployed	2	0.9	0	0	2	1.6
Missing data	26	12.1	10	11.1	16	12.8
Performance Status at baseline						
0	192	89.3	81	90	111	88.8
1	13	6.0	4	4.4	9	7.2
2	2	0.9	1	1.1	1	0.8
3	0	0	0	0	0	0
4	0	0	0	0	0	0
Missing data	8	3.7	4	4.4	4	3.2
Cancer stage at diagnosis						
Local	205	95.3	84	93.3	121	96.8
Locoregional	6	2.8	2	2.2	4	3.2
Metastatic	0	0	0	0	0	0
Missing data	4	1.9	4	4.4	0	0
Disease treatment						
Chemotherapy						
Yes	0		0	0	0	0
No	208	96.7	86	95.6	122	97.6
Missing data	7	3.3	4	4.4	3	2.4
Radiotherapy						
Yes	112	52.1	58	64.4	54	43.2
No	97	45.1	27	21.4	70	56
Missing data	6	2.8	5	5.6	1	0.8
Hormone therapy						
Yes	45	20.9	22	17.5	23	18.4
No	162	75.3	64	71.1	98	78.4
Missing data	8	3.7	4	4.4	4	3.2
Surgery						
Yes	103	47.9	44	48.9	59	47.2
No	103	47.9	41	45.5	62	49.6
Missing data	9	4.2	5	5.6	4	3.2
Brachytherapy						
Yes	17	7.9	13	14.4	4	3.2
No	187	87.0	73	81.1	114	91.2
Missing data	11	5.1	4	4.4	7	5.6

#### Validation of the psychometric properties

##### Acceptability

Nineteen patients (8.8%) did not fill out the baseline EPIC questionnaire: 11 patients (8.8%) in the Treatment group and 8 patients (8.8%) in the Cured group. Nine of these patients (47.4%) reported that they forgot to return the questionnaire. Eighty-one patients (37.7%) fully completed the baseline questionnaire.

##### Floor and ceiling effects

Mean (SD) scores for all patients at baseline, as well as the percentage of lowest or highest possible scores to quantify floor and ceiling effects respectively, for each subscale and for each summary score are given in Table 2. A ceiling effect (i.e., more than 15% of patients having the highest possible score) was observed for the bowel (15.35%) and hormonal (20.93%) summary scores, and for the main subscales, except for the sexual subscales, bother, and the irritation/obstruction subscales of the urinary domain. No floor effect was observed.

**Table 2:** Characteristics of EPIC domain-specific summary and subscales scores (*N* = 215; T_1_)

HRQoL domain	Number of Items	*N*	mean score (SD)	range (min-max)	Floor effects (%)	Ceiling effects (%)	Cronbach’s α	Cronbach’s α in the original version (Wei et al. 2000)
HRQoL Domain Summary Scores								
Urinary	12	167	86.74 (13.61)	33.33–100	0	9.30	0.88	0.88
Bowel	14	165	91.70 (9.93)	30.36–100	0	15.35	0.61	0.92
Sexual	13	177	43.88 (21.98)	0.0–94.23	0.93	0	0.89	0.93
Hormonal	11	162	87.67 (12.49)	47.50–100	0	20.93	0.77	0.82
Domain-Specific HRQoL Subscales								
*Urinary subscales*								
Function	5	192	94.39 (11.02)	40.00–100	0	60.47	0.63	0.69
Bother	7	162	82.03 (17.33)	17.86–100	0	10.70	0.85	0.85
Irritation/obstruction	7	163	85.42 (14.42)	17.86–100	0	11.16	0.79	0.81
Incontinence	4	167	91.44 (17.22)	22.75–100	0	53.93	0.91	0.89
*Bowel subscales*								
Function	7	190	92.46 (9.07)	39.29–100	0	23.72	0.53	0.75
Bother	7	165	90.72 (13.36)	21.43–100	0	30.70	0.83	0.90
*Sexual subscales*								
Function	9	174	40.76 (28.96)	0.00–91.67	8.84	0	0.96	0.92
Bother	4	166	53.61 (34.14)	0.00–100	7.91	14.88	0.94	0.84
*Hormonal subscales*								
Function	5	186	86.99 (13.18)	25.00–100	0	26.51	0.76	0.51
Bother	6	163	88.32 (13.59)	45.83–100	0	25.58	0.63	0.73

##### Reliability

Regarding internal consistency, Cronbach’s alpha was ≥ 0.70 for each subscale except for urinary function, bowel function and hormonal symptoms, where Cronbach’s alpha was 0.63, 0.53 and 0.63 respectively (Table 2). Regarding the summary scores, Cronbach’s alpha coefficient was <0.70 for the bowel domain only (0.61).

Table 3 displays the mean (SD) scores for each summary score and subscale for patients in the Cured group at the two measurement times, as well as the ICC for test-retest reliability. An ICC ≥ 0.70 was observed for each subscale and summary score, except for bowel function, where the ICC was 0.68.

**Table 3:** Assessment of test-retest reliability between the two assessment times for the Cured group

	Time 1: baseline		Time 2: two weeks later		
HRQoL domain	*N*	mean (SD)	*N*	mean (SD)	ICC for test-retest (ICC of Original version)
HRQoL domain summary scores					
Urinary	71	84.31 (15.01)	64	86.19 (14.18)	0.90 (0.88)
Bowel	70	89.34 (11.79)	67	89.54 (12.64)	0.86 (0.84)
Sexual	72	33.18 (20.84)	73	33.67 (20.18)	0.94 (0.91)
Hormonal	70	86.70 (13.15)	67	88.53 (13.34)	0.89 (0.80)
Domain-specific HRQoL subscales					
*Urinary subscales*					
Function	80	90.73 (14.37)	76	91.80 (13.29)	0.84 (0.83)
Bother	68	80.42 (17.11)	63	82.36 (18.39)	0.90 (0.87)
Irritation/obstruction	68	85.31 (13.28)	63	86.43 (13.48)	0.82 (0.85)
Incontinence	72	85.10 (22.09)	65	87.88 (19.28)	0.94 (0.87)
*Bowel subscales*					
Function	80	90.76 (10.46)	77	91.79 (9.94)	0.68 (0.78)
Bother	70	88.10 (15.14)	66	88.49 (16.26)	0.88 (0.85)
*Sexual subscales*					
Function	71	26.06 (26.82)	74	25.07 (25.80)	0.98 (0.90)
Bother	68	53.03 (36.36)	69	51.90 (33.23)	0.78 (0.78)
*Hormonal subscales*					
Function	78	85.40 (15.07)	76	88.49 (13.63)	0.76 (0.79)
Bother	70	87.50 (13.63)	65	89.91 (13.44)	0.84 (0.73)

*SD* standard deviation, *ICC* intraclass coefficient

##### Construct validity

Convergent validity was achieved for all items of the sexual function subscale, with a correlation between the sexual function score and summary score greater than 0.4 (Table 4). Regarding the items of the sexual bother subscale, the convergent validity was respected in terms of correlation with the sexual bother score, but not for the sexual domain summary score, with the correlation between each item and the summary score being less than 0.4. Convergent validity was also respected for all items of the urinary bother subscale, except for item q30, with a correlation of −0.28 with both the urinary bother subscale score and the urinary domain summary score. Likewise, convergent validity was also respected for all items of the bowel bother subscale, except for item q53, with a correlation of −0.38 with the bowel bother subscale score, and −0.31 with bowel domain summary score. For all other dimensions, the convergent validity was respected for only a minority of items, in particular hormonal function, where no item of this subscale respected the convergent validity with respect to its own subscale. Yet, most of items of the hormonal scale respected the convergent validity with the overall hormonal domain.

**Table 4:** Mutitrait matrix with correlation of each item and the domain-specific HRQoL subscales and summary scores

Domain	Subscale	Items	Urinary domain	Urinary Function	Urinary Bother	Bowel domain	Bowel Function	Bowel Bother	Sexual domain	Sexual Function	Sexual Bother	Hormonal domain	Hormonal Function	Hormonal Bother
Urinary	Function	**q23**	**0.48**	**0.59**	0.56	0.38	0.27	0.36	0.29	0.28	0.06	0.32	0.30	0.29
		**q24**	0.15	−0.04	0.21	0.16	0.06	0.19	0.03	0.03	0.02	0.03	−0.03	0.08
		**q25**	**0.43**	0.21	0.40	0.42	0.41	0.32	0.11	0.11	0.01	0.22	0.20	0.16
		**q26**	**0.58**	**0.63**	0.50	0.43	0.32	0.39	0.32	0.33	0.02	0.27	0.31	0.21
		**q27**	**−0.50**	**−0.63**	−0.40	−0.31	−0.30	−0.24	−0.26	−0.25	−0.07	−0.37	−0.40	−0.30
	Bother	**q28**	**−0.60**	**−0.72**	**−0.45**	−0.34	−0.30	−0.30	−0.29	−0.26	−0.10	−0.33	−0.33	−0.29
		**q29**	**−0.54**	−0.46	**−0.50**	−0.48	−0.36	−0.46	−0.13	−0.08	−0.11	−0.32	−0.25	−0.30
		**q30**	−0.28	−0.19	−0.28	−0.19	−0.01	−0.25	−0.03	−0.03	−0.03	−0.23	−0.14	−0.27
		**q31**	**−0.58**	−0.35	**−0.62**	−0.46	−0.35	−0.45	−0.17	−0.12	−0.14	−0.32	−0.26	−0.32
		**q32**	**−0.59**	−0.32	**−0.65**	−0.42	−0.32	−0.37	−0.25	−0.26	−0.07	−0.37	−0.32	−0.36
		**q33**	**0.56**	−0.32	**−0.60**	−0.33	−0.27	−0.27	−0.26	−0.23	−0.13	−0.29	−0.21	−0.28
		**q34**	**−0.75**	−0.52	**−0.74**	−0.43	0.31	−0.42	−0.27	−0.24	−0.11	−0.39	−0.36	−0.36
Bowel	Function	**q42**	0.23	0.23	0.22	0.28	0.24	0.27	0.16	0.16	0.02	0.13	0.20	0.12
		**q43**	0.31	0.40	0.25	**0.50**	0.34	**0.51**	0.10	0.06	0.05	0.33	0.29	0.30
		**q44**	−0.25	−0.14	−0.25	−0.32	−0.23	−0.32	−0.08	−0.10	0.03	−0.28	−0.20	−0.30
		**q45**	−0.15	−0.13	−0.14	−0.17	−0.17	**−0.18**	−0.17	**−0.19**	0.02	−0.10	−0.11	−0.10
		**q46**	−0.36	−0.34	−0.33	**−0.48**	**−0.40**	**−0.45**	−0.23	−0.20	−0.08	−0.30	−0.26	−0.29
		**q47**	−0.11	−0.12	−0.11	−0.19	−0.17	**−0.19**	−0.09	−0.10	0.02	−0.06	−0.12	−0.02
		**q48**	0.38	0.30	0.33	**0.49**	**0.42**	**0.44**	0.10	0.05	0.07	0.13	0.23	0.07
	Bother	**q49**	−0.42	−0.35	−0.39	**−0.69**	−0.51	**−0.62**	−0.27	−0.24	−0.08	−0.3	−0.22	−0.34
		**q50**	−0.49	−0.44	−0.48	**−0.67**	−0.52	**−0.64**	−0.29	−0.24	−0.14	−0.36	−0.28	−0.37
		**q51**	−0.30	−0.18	−0.34	**−0.53**	−0.36	**−0.55**	−0.16	−0.19	−0.15	−0.24	−0.15	−0.30
		**q52**	−0.39	−0.39	−0.35	**−0.56**	−0.38	**−0.60**	−0.06	0.01	−0.09	−0.27	−0.19	−0.30
		**q53**	−0.35	−0.27	−0.36	−0.31	**−0.40**	−0.38	−0.09	−0.02	−0.12	−0.24	−0.17	−0.28
		**q54**	−0.35	−0.27	−0.36	**−0.57**	−0.40	**−0.55**	−0.09	−0.02	−0.12	−0.24	−0.17	−0.28
		**q55**	−0.37	−0.32	−0.33	**−0.61**	−0.50	**−0.58**	−0.21	−0.13	−0.18	−0.36	−0.26	−0.37
Sexual	Function	**q56**	0.28	0.30	0.22	0.23	0.24	0.15	**0.56**	**0.76**	−0.23	0.14	0.22	0.10
		**q57**	0.28	0.32	0.23	0.25	0.22	0.20	**0.83**	**0.89**	0.09	0.12	0.18	0.10
		**q58**	0.30	0.29	0.26	0.22	0.22	0.17	**0.72**	**0.86**	−0.04	0.15	0.20	0.13
		**q59**	0.33	0.37	0.27	0.25	0.22	0.20	**0.71**	**0.83**	−0.06	0.14	0.17	0.14
		**q60**	0.29	0.32	0.24	0.24	0.19	0.17	**0.76**	**0.85**	0.02	0.09	0.11	0.08
		**q61**	0.33	0.29	0.31	0.25	0.27	0.18	**0.52**	**0.60**	−0.05	0.14	0.19	0.14
		**q62**	0.27	0.28	0.22	0.22	0.20	0.17	**0.69**	**0.80**	−0.04	0.11	0.16	0.09
		**q63**	0.22	0.27	0.17	0.19	0.18	0.14	**0.69**	**0.83**	−0.07	0.06	0.11	0.05
		**q64**	0.23	0.26	0.20	0.24	0.20	0.17	**0.79**	**0.91**	−0.02	0.07	0.11	0.05
	Bother	**q65**	−0.11	<0.01	−0.14	−0.12	−0.04	−0.16	−0.22	0.09	**−0.90**	−0.23	−0.09	−0.24
		**q66**	−0.13	−0.04	−0.16	−0.13	−0.05	−0.16	−0.27	0.06	**−0.91**	−0.25	−0.14	−0.26
		**q67**	−0.09	−0.05	−0.08	−0.08	<0.01	−0.14	−0.25	0.07	**−0.88**	−0.27	−0.13	−0.29
		**q68**	−0.19	−0.07	−0.22	−0.16	−0.07	−0.18	−0.30	−0.04	**−0.75**	−0.23	−0.18	−0.20
Hormonal	Function	**q69**	**0.16**	**0.16**	**0.16**	**0.13**	**0.20**	0.05	0.22	0.28	−0.05	0.31	0.11	0.36
		**q70**	**0.23**	0.12	0.22	0.01	0.19	0.01	−0.10	0.01	−0.05	0.15	0.22	0.09
		**q71**	0.20	0.21	0.22	**0.26**	0.20	**0.25**	0.10	−0.02	**0.24**	**0.46**	0.23	**0.53**
		**q72**	0.40	0.29	0.37	0.31	0.23	0.28	0.17	0.07	0.21	**0.51**	0.34	**0.54**
		**q73**	−0.08	−0.04	−0.05	0.08	0.08	0.06	0.04	0.06	−0.06	0.03	0.08	−0.02
	Bother	**q74**	−0.19	−0.11	−0.19	−0.09	−0.12	−0.03	−0.20	−0.16	−0.15	**−0.43**	−0.48	−0.27
		**q75**	**−0.25**	−0.09	−0.30	−0.10	−0.16	−0.09	0.03	0.05	<0.01	**−0.11**	−0.21	−0.05
		**q76**	−0.17	−0.07	−0.20	−0.12	−0.08	−0.15	0.05	0.10	0.01	**−0.17**	−0.05	−0.25
		**q77**	−0.30	−0.23	−0.28	−0.40	−0.26	−0.45	−0.10	0.02	−0.26	**−0.58**	−0.54	**−0.54**
		**q78**	−0.40	−0.32	−0.37	−0.41	−0.30	−0.42	−0.21	−0.08	−0.31	**−0.64**	−0.58	**−0.60**
		**q79**	−0.24	−0.16	**−0.28**	−0.23	−0.15	−0.23	−0.17	−0.10	−0.18	−0.33	−0.40	−0.23

Values in bold for item own-scale correlation and item own-domain correlation correspond to items respecting convergent validity, i.e., correlation >0.4 in absolute value. Values in bold for correlation between an item and other scale scores correspond to items that did not respect discriminant validity, i.e., correlation between the item and the other scale scores was greater than that between the item and its own-scale score

Discriminant validity was achieved for all items of both the urinary and sexual domains. Conversely, discriminant validity was not respected for 5/7 items of the bowel function subscale, 1/7 item of the bowel bother subscale, 3/5 items of the hormonal function subscale and 2/6 items of the hormonal bother subscale.

The result of the EFA is summarized in Table 5. Fifty-three percent of the variance was explained by the four factors. Items of the sexual function subscale were highly correlated with Factor 1, while those of the sexual bother subscale were correlated with Factor 2. Most of the items of the urinary domain were correlated with Factor 3 while those of the bowel domain were correlated with Factor 4. Finally, items of the hormonal domain were equally distributed over the first three factors.

**Table 5:** Exploratory factor analysis with varimax rotation and 4 factors performed at the first measurement time including all patients with complete data (*N* = 111)

Domain	Subscale	Item	Factor 1	Factor 2	Factor 3	Factor 4
Urinary	Function	**q23**	0.164	−0.141	**−0.676**	−0.058
		**q24**	−0.017	−0.036	0.097	**−0.294**
		**q25**	0.022	0.075	−0.117	−0.583
		**q26**	0.180	−0.024	**−0.709**	−0.159
		**q27**	−0.172	0.065	**0.700**	0.022
	Bother	**q28**	−0.128	0.048	**0.836**	−0.004
		**q29**	0.013	0.051	0.129	**0.690**
		**q30**	0.142	0.098	0.129	**0.245**
		**q31**	−0.009	0.135	**0.502**	0.323
		**q32**	−0.091	0.237	**0.467**	0.351
		**q33**	−0.182	0.149	**0.505**	0.176
		**q34**	−0.121	0.206	**0.617**	0.304
Bowel	Function	**q42**	0.167	0.059	0.001	**−0.304**
		**q43**	−0.064	−0.232	**−0.410**	−0.064
		**q44**	−0.114	0.114	0.151	**0.265**
		**q45**	−0.144	0.005	**0.240**	−0.055
		**q46**	−0.191	0.096	−0.038	**0.398**
		**q47**	−0.149	−0.025	−0.041	**0.277**
		**q48**	−0.063	0.056	−0.185	**−0.586**
	Bother	**q49**	−0.175	0.164	0.148	**0.579**
		**q50**	−0.129	0.107	0.264	**0.557**
		**q51**	0.008	0.216	−0.017	**0.504**
		**q52**	0.145	0.192	0.234	**0.483**
		**q53**	0.021	0.184	0.076	**0.384**
		**q54**	0.098	0.066	−0.028	**0.765**
		**q55**	−0.078	0.326	0.058	**0.384**
Sexual	Function	**q56**	**0.820**	0.097	−0.056	0.007
		**q57**	**0.909**	−0.101	−0.056	0.024
		**q58**	**0.885**	0.002	−0.077	0.005
		**q59**	**0.846**	0.008	−0.127	0.012
		**q60**	**0.884**	−0.093	−0.060	0.005
		**q61**	**0.604**	0.037	−0.143	−0.065
		**q62**	**0.791**	0.092	−0.099	−0.027
		**q63**	**0.838**	0.134	−0.053	−0.005
		**q64**	**0.923**	−0.041	0.037	−0.074
	Bother	**q65**	0.022	**0.831**	0.099	−0.013
		**q66**	−0.013	**0.838**	0.045	0.029
		**q67**	0.017	**0.841**	0.058	0.007
		**q68**	−0.047	**0.735**	0.067	0.041
Hormonal	Function	**q69**	**0.260**	−0.087	−0.239	0.032
		**q70**	−0.104	0.056	**−0.412**	0.151
		**q71**	−0.052	**−0.646**	0.024	−0.074
		**q72**	0.071	**−0.564**	−0.179	−0.065
		**q73**	0.074	−0.034	**0.249**	0.060
	Bother	**q74**	−0.157	0.279	0.270	−0.101
		**q75**	0.131	−0.046	**0.443**	−0.119
		**q76**	**0.212**	0.057	0.054	0.141
		**q77**	0.112	**0.662**	0.020	0.256
		**q78**	−0.018	**0.636**	0.161	0.137
		**q79**	−0.019	0.159	0.266	0.133

Values in bold correspond to the highest correlation observed between each item and factors

##### Criterion validity

Correlation between each EPIC summary score and the equivalent scale of the QLQ-PR25 was greater than 0.4, highlighting good criterion validity (Table 6). In particular, the correlation was highest between the urinary symptoms scale of the QLQ-PR25 module and the urinary summary score of the EPIC questionnaire, at −0.83. The urinary, sexual and hormonal summary scores of the EPIC were also highly correlated with the incontinence aid dimension of the QLQ-PR25 (at −0.66, −0.55 and −0.57, respectively).

**Table 6:** Interscale correlations between EPIC summary scores and subscales and QLQ-PR25 scores (*N* = 215; T1)

EPIC	Urinary					Bowel			Sexual			Hormonal		
	Domain	Function	Bother	Irritation Obstruction	Incontinence	Domain	Function	Bother	Domain	Function	Bother	Domain	Function	Bother
QLQ-PR25														
Sexual activity	−0.13	−0.13	−0.12	−0.12	−0.10	−0.09	−0.15	−0.07	**−0.48**	**−0.65**	**0.17**	−0.08	−0.15	−0.05
Sexual functioning	−0.21	−0.20	−0.20	−0.16	−0.23	−0.25	−0.16	−0.25	**−0.62**	**−0.62**	**−0.20**	−0.15	−0.23	−0.14
Urinary symptoms	**−0.83**	**−0.62**	**−0.81**	**−0.76**	**−0.59**	−0.58	−0.51	−0.48	−0.32	−0.31	−0.06	−0.38	−0.40	−0.34
Bowel symptoms	−0.44	−0.45	−0.39	−0.34	−0.41	**−0.65**	**−0.54**	**−0.62**	−0.28	−0.23	−0.12	−0.36	−0.28	−0.35
Hormonal treatment-related symptoms	−0.33	−0.23	−0.37	−0.30	−0.24	−0.33	−0.34	−0.27	−0.40	−0.36	−0.17	**−0.52**	**−0.45**	**−0.50**
Incontinence aid	−0.66	−0.61	−0.60	−0.30	**−0.65**	−0.27	−0.38	−0.10	−0.55	−0.43	−0.53	−0.57	−0.58	−0.40

Values in bold correspond to correlation between EPIC domains and subscales and the equivalents scales of the QLQ-PR25. For the QLQ-PR25 a high score indicates a high functional level and a high symptomatic level. For the EPIC questionnaire, a high score indicates a high health-related quality of life level

##### Sensitivity to change

The median time between the two assessments was 7.8 weeks (range 3.2–12.8). Among the patients of the Treatment group, 55 (44%) experienced at least one toxicity rated grade 2 or higher during treatment. For all subscales and summary scores, these patients experienced a decrease of mean HRQoL at T2 as compared to T1, with a clinically significant difference (10-point MCID) observed for 7/10 subscales and 2/4 summary scores (Table 7). As compared to patients without toxicity during treatment, patients who experienced toxicities presented a greater decrease in the sexual summary score (mean difference = −30.59 vs. -13.61, *P*-value < 0.001), urinary function subscale (mean difference = −31.57 vs. -18.48, *P*-value = 0.004), incontinence subscale (mean difference = −45.67 vs. -18.13, *P*-value < 0.001), sexual function subscale (mean difference = −38.68 vs. -21.80, *P*-value = 0.001) and sexual bother subscale (mean difference = −14.10 vs. 1.83, *P*-value = 0.022).

**Table 7:** Mean difference between the two assessments (T2 minus T1) for Treatment group patients, comparing patients without toxicities with patients who experienced toxicities during treatment, in order to assess sensitivity to change

	Patients without toxicities (*N* = 70)			Patients experienced toxicities (*N* = 55)			
Dimensions	*N*	mean difference (SD)	SRM	*N*	mean difference (SD)	SRM	*P*-value
HRQoL domain summary scores							
Urinary	41	−18.49 (22.80)	−0.81	43	−27.64 (19.44)	−1.42	0.051
Bowel	40	−11.30 (17.15)	−0.66	39	−9.16 (12.16)	−0.75	0.522
Sexual	48	−13.61 (17.72)	−0.77	44	−30.59 (24.42)	−1.25	<0.001
Hormonal	36	−6.48 (13.67)	−0.47	41	−5.29 (10.24)	−0.52	0.664
Domain-specific HRQoL subscales							
*Urinary subscales*							
Function	52	−18.48 (22.57)	−0.82	47	−31.57 (21.21)	−1.49	0.004
Bother	39	−20.25 (24.54)	−0.83	41	−24.03 (24.01)	−1.00	0.489
Irritation/obstruction	40	−16.96 (23.39)	−0.73	41	−15.90 (21.00)	−0.76	0.830
Incontinence	41	−18.13 (29.86)	−0.61	43	−45.67 (31.66)	−1.44	<0.001
*Bowel subscales*							
Function	51	−11.03 (17.15)	−0.64	48	−10.79 (15.21)	−0.71	0.941
Bother	39	−9.20 (17.01)	−0.54	39	−6.01 (15.49)	−0.39	0.389
*Sexual subscales*							
Function	47	−21.80 (23.42)	−0.93	44	−38.68 (24.32)	−1.59	0.001
Bother	41	1.83 (24.34)	0.07	43	−14.10 (37.05)	−0.38	0.022
*Hormonal subscales*							
Function	49	−5.13 (14.10)	−0.36	46	−7.42 (15.61)	−0.47	0.454
Bother	38	−6.64 (14.89)	−0.45	41	−4.27 (10.75)	−0.40	0.422

*SD* standard deviation, *SRM* standardized Response Mean; *P*-value of paired *T*-test

## Discussion

This paper reports the cross-cultural adaptation and validation of the French version of the EPIC HRQoL questionnaire specific to prostate cancer, using classical test theory. The qualitative step overall exhibited good acceptability and understanding of the questionnaire. Few patients reported that they found the questions to be disturbing, and the comments provided showed that this was not due to translation problems, but rather to the construction of the questionnaire itself, since some symptoms were not experienced by the patients and therefore, they found it difficult to judge its impact, while some other items were perceived to be too private. The number of patients who refused to participate in this study could also be informative for acceptability, but unfortunately, it was not possible to collect this data in each center.

Ceiling effects were found for bowel and urinary summary scores, although the percentage of patients with the highest possible score was close to the threshold of 15% for the urinary domain. A high ceiling effect was observed for the function and incontinence subscales of the urinary domain. Conversely, no floor effect was observed for any domain or subscale. Similar findings were observed in the original English version of the EPIC questionnaire [12].

Good internal consistency was observed for all summary scores, except for the bowel domain, where the Cronbach’s alpha was 0.61. The repeatability was also demonstrated for all domains and subscales, expect for bowel function (ICC = 0.68). Thus, the reliability of the questionnaire was globally good for all scales, except for the bowel dimension. In comparison, good internal consistency and repeatability were observed for each subscale, except for hormonal function (Cronbach’s alpha coefficient 0.51), and for all summary scores in the original English version [12].

We also observed high criterion validity, with a high correlation between EPIC domains and the equivalent scales of the QLQ-PR25. Wei et al. [12] compared the English version of the EPIC questionnaire to both the Functional Assessment of Cancer Therapy-Prostate (FACT-P) module [24] and to the American Urological Association Symptom Index (AUA-SI) [25, 26]. For both these prostate-cancer-specific questionnaires, only one overall score is generated. The QLQ-PR25 presents the advantage, as with the EPIC, of generating scores for each HRQoL domain, and may thus be considered to be more precise.

The sensitivity to change assessed in this study highlighted a clinically and statistically significant change on 5 of the 8 subscales, and 2 of the 4 summary scores in patients who experienced toxicity during treatment. Compared to patients who did not suffer toxicity during treatment, those with toxicities had a greater decrease in the sexual summary score, and in the urinary and sexual subscales. Wei et al. did not assess the sensitivity to change of the original English version of the EPIC, therefore precluding comparison with our results [12].

Regarding the construct validity, multitrait analysis and EFA showed complementary results. Only the urinary domain and sexual function subscale reached the goal of good construct validity. Most of the dimensions showed good discriminant validity, except for bowel and hormonal functions. The bowel bother subscale also exhibited good convergent validity. The sexual function and bother subscales seemed to capture complementary results, since a bidimensional component was highlighted by the EFA. In fact, these subscales were highly correlated with two separate factors in the EFA. Moreover, items of the sexual bother subscale were poorly correlated to the sexual summary scores, as shown by the multitrait analysis. The hormonal domain presented poor construct validity overall, since neither convergent nor discriminant validity was respected, and correlation was observed with three of the four factors of the EFA. These poor results for the hormonal domain could be partially explained by the low number of patients treated by hormone therapy (only 45 patients (20.9%)). The construct validity was not reported for the original English version of the EPIC questionnaire, rendering comparison impossible. These poor results could also be partially explained by the low number of patients with complete data included in the EFA analysis (only 111 patients), whereas at least 150 patients are required to ensure the robustness of these analyses [18]. Indeed, the main limitation of this study is the low number of patients involved in step 2. Only 215 patients were actually enrolled in step 2, whereas a sample size of 300 patients was initially expected. Nonetheless, this sample size may be sufficient for most of the statistical analyses, even though the observed statistical power was lower than expected. The low sample size stems from the fact that the inclusion period was longer than planned, and the study had to be interrupted due to financial constraints.

One of the strengths of this study is the assessment of all psychometric properties, including both reproducibility and sensitivity to change, which are not always systematically assessed in the validation process of HRQoL questionnaires. In order to complement these results according to classical test theory, particularly the construct validity, a modern psychometric approach using item response theory is essential [27], such as the use of Rasch-family models [28, 29]. These analyses are planned and will be fully reported in a separate paper. Moreover, these analyses present the advantage of being robust in the presence of missing data, and thus, all patients with at least one item completed can contribute to the analyses [30]. These further analyses should make it possible to validate or rule out the trend observed on EFA analysis.

It would also be interesting to study the impact of other demographic and clinical data on the results of the EPIC validation, including the impact of age, relationship status or treatment modality and race. However, the sample size was not sufficient to ensure adequate variability to enable these additional analyses. We also plan to study the occurrence of differential item functioning using item response theory analysis. While the impact of race could be of interest, in France, as in several other European countries, current legislation does not allow data on racial origin to be collected routinely in studies, without ample justification of the importance of this variable.

Another strength of this study is that both patients with ongoing treatment and cured patients were included in the validation process. This enables validation of the EPIC questionnaire for future epidemiological studies performed late after diagnosis.

This adaptation and validation of the French version of the EPIC questionnaire was essential in order to enable the use this tool in future French-language studies. This validation was also mandatory to enable the second part of the QALIPRO study, which aims to investigate the long-term side effects of prostate cancer treatment. Moreover, although this questionnaire was initially developed for prostate cancer patients, other elderly patients without prostate cancer could be concerned by some of the issues mentioned in this instrument. In fact, in the debriefing questionnaire in step 1, forty-one patients (91.1%) considered that the questionnaire could concern other men with no prostate cancer.

## Conclusions

In conclusion, the French version of the EPIC questionnaire showed good psychometric properties in patients with prostate cancer, similar to those of the original English version. An item response theory analysis will complete these results.

## Abbreviations

AUA-SI: American Urological Association Symptom Index
EFA: Exploratory factor analysis
EORTC: European Organisation of Research and Treatment of Cancer
EPIC: Expanded prostate cancer index composite
ES: Effect size
FACT-P: Functional assessment of cancer therapy-prostate
GHS: Global health status
HRQoL: Health-related quality of life
ICC: Interclass correlation coefficient
MCID: Minimal clinically important difference
SD: Standard deviation
SRM: Standardized response mean
UCLA-PCI: University of California-Los Angeles Prostate Cancer Index
